# Prognostic Value of Dehydroepiandrosterone-Sulfate and Other Parameters of Adrenal Function in Acute Ischemic Stroke

**DOI:** 10.1371/journal.pone.0063224

**Published:** 2013-05-01

**Authors:** Claudine A. Blum, Cornelia Mueller, Philipp Schuetz, Felix Fluri, Michael Trummler, Beat Mueller, Mira Katan, Mirjam Christ-Crain

**Affiliations:** 1 Department of Endocrinology, Medical University Clinic, Cantonal Hospital, Aarau, Switzerland; 2 Endocrinology, Diabetology and Metabolism, University Hospital, Basel, Switzerland; 3 Division of Neurology, University Hospital Basel, Basel, Switzerland; 4 Clinic of Neurology, University Hospital Zurich, Zürich, Switzerland; 5 Bioanalytica AG, Luzern, Switzerland; Georgia Health Sciences University, United States of America

## Abstract

**Background and Purpose:**

Acute stroke has a high morbidity and mortality. We evaluated the predictive value of adrenal function testing in acute ischemic stroke**.**

**Methods:**

In a cohort of 231 acute ischemic stroke patients, we measured dehydroepiandrosterone (DHEA), DHEA-Sulfate (DHEAS), cortisol at baseline and 30 minutes after stimulation with 1 ug ACTH. Delta cortisol, the amount of rise in the 1 ug ACTH-test, was calculated. Primary endpoint was poor functional outcome defined as modified Rankin scale 3–6 after 1 year. Secondary endpoint was nonsurvival after 1 year.

**Results:**

Logistic regression analysis showed that DHEAS (OR 1.21, 95% CI 1.01–1.49), but not DHEA (OR 1.01, 95% CI 0.99–1.04), was predictive for adverse functional outcome. Neither DHEA (OR 0.99, 95% CI 0.96–1.03) nor DHEAS (OR 1.10, 95% CI 0.82–1.44) were associated with mortality. Baseline and stimulated cortisol were predictive for mortality (OR 1.41, 95% CI 1.20–1.71; 1.35, 95% CI 1.15–1.60), but only basal cortisol for functional outcome (OR 1.20, 95% CI 1.04–1.38). Delta cortisol was not predictive for functional outcome (OR 0.86, 95% CI 0.71–1.05) or mortality (OR 0.92, 95% CI 0.72–1.17). The ratios cortisol/DHEA and cortisol/DHEAS discriminated between favorable outcome and nonsurvival (both p<0.0001) and between unfavorable outcome and nonsurvival (p = 0.0071 and 0.0029), but are not independent predictors for functional outcome or mortality in multivariate analysis (adjusted OR for functional outcome for both 1.0 (95% CI 0.99–1.0), adjusted OR for mortality for both 1.0 (95% CI 0.99–1.0 and 1.0–1.01, respectively)).

**Conclusion:**

DHEAS and the cortisol/DHEAS ratio predicts functional outcome 1 year after stroke whereas cortisol levels predict functional outcome and mortality.

**Trial Registration:**

ClinicalTrials.gov NCT00390962 (Retrospective analysis of this cohort).

## Introduction

Stroke is the third-leading cause for disability worldwide [1] with an incidence of about 500 per 100’000 persons at the age of 60 and a disease-related mortality of 20% [2].Therefore, early risk stratification for an optimized allocation of health care resources is warranted.

Activation of the hypothalamo-pituitary-adrenal (HPA)-axis has been shown in various acute critical illnesses [3], [4]. It is one of the first measurable physiological responses to cerebral ischemia [5]–[8] and cortisol levels are predictive of functional outcome in acute stroke [9]–[11]. Besides cortisol, dehydroepiandrosterone (DHEA) and its sulfate (DHEAS) are also released during HPA-activation. DHEAS is the most abundant steroid of the adrenals. Under healthy condition, DHEA secretion is synchronized with cortisol in response to corticotrophin-releasing hormone and ACTH [12], [13]. A dysbalance or inadequate stress response with down-regulation of DHEAS is associated with an unfavorable outcome in severe critical illness, severe sepsis and septic shock in some, but not all studies [14]–[16].

DHEAS has antiglucocorticoid activity, neuroprotective and antiatherosclerotic properties [17]–[22]. In rodents, synthesis of DHEA and DHEAS has been shown in the brain [23]–[25]. In addition, central nervous system DHEA production seems to influence peripheral DHEA and DHEAS levels [26]. In longitudinal studies, an increased cortisol/DHEAS ratio has been found to accelerate atherosclerosis-related diseases [27] and to be predictive for cardiovascular diseases [28] and all-cause-mortality [29]. In chronic stress [30] and neurodegenerative diseases [31]–[33], higher cortisol and lower serum DHEA and DHEAS values with a consecutive higher cortisol/DHEAS-ratio have been found. In the acute setting, high cortisol and an increased cortisol/DHEAS – ratio upon admission is associated with severity of illness in intensive care patients [34], corresponding to an impaired adrenal androgen action [35].

In acute ischemic stroke, only two studies so far investigated the predictive role of serum DHEAS, with controversial findings; one [5] did not find a significant correlation between DHEAS and functional outcome. The other study including only women found a significant association between DHEAS and functional outcome [36].

In critical illness, an impairment of secretion of basal cortisol and the corticosteroid response to ACTH, is a highly debated topic [37], but few and conflicting data exist in stroke about the predictive value of the ACTH-test [38], [39]. In view of the controversial findings about DHEA, DHEAS and the low-dose (1 µg) ACTH-test as outcome predictors in ischemic stroke, we herein evaluated the predictive value of adrenal function testing in a cohort of prospectively recruited stroke patients [8] by measuring DHEA, DHEAS, basal and stimulated cortisol levels.

## Subjects and Methods

### Study Design and Setting

The study design of this prospective cohort study has been described in detail [8]. From November 2006 to November 2007, consecutive patients presenting with acute ischemic stroke were enrolled. Informed consent was obtained from the patient if possible, otherwise from a legal representative. This study adhered to the consolidate standards for the reporting of observational trials [40] and was accepted by the local ethics committee.

### Patients, Clinical Variables, Blood Sampling and Cerebral Imaging

Patients were eligible for inclusion into the original study [8] if they were admitted to the emergency department with an acute ischemic stroke defined according to World Health Organization criteria [41] and with symptom onset within 72 hours. For the purpose of this analysis, we included only patients in whom a low-dose-ACTH- test (Synacthen©) had been performed on day 1 (i.e. the first morning after admission) according to study protocol and a remaining serum sample was available from the same day. Delta cortisol was calculated as the difference between basal and stimulated cortisol level.

Vital signs, relevant comorbidities, medication before stroke, risk factors, family history and severity of stroke assessed by The National Institutes of Health Stroke Scale (NIHSS) score were assessed on admission. Cerebral computed tomography (CCT) was performed in all patients on admission mainly to exclude intracranial hemorrhage. Thereafter, MRI was performed on a clinical 1.5-Tesla MR Avanto system (Siemens, Erlangen, Germany) using a stroke protocol, including T1-, T2-, and diffusion-weighted imaging (DWI) sequences, and a magnetic resonance angiography. MRI with DWI was available in 137 stroke patients (59%). In those patients, DWI lesion volumes were determined by consensus of two experienced raters unaware of the clinical and laboratory results.

The clinical stroke syndrome was determined applying the criteria of the Oxfordshire Community Stroke Project, that is, total anterior circulation syndrome (TACS), partial anterior circulation syndrome (PACS), lacunar syndrome (LACS),and posterior circulation syndrome (POCS) [42].

### Hypothesis and Endpoints

We hypothesized that activation of the HPA-axis with consecutive dysbalance of adrenal steroids is associated with functional outcome and mortality in acute stroke, similar to the associations seen in critical illness.

The primary endpoint was poor functional outcome defined as a modified Rankin scale (mRS) of 3–6 points [43]. The secondary endpoint was death (mRS = 6). Both endpoints were assessed by structured telephone interviews with the patient, relatives and/or treating primary care physician one year after the index event.

### Assays

Blood samples were obtained from an indwelling venous catheter between 7 and 8 a.m. in the morning after admission. Plasma was collected at the time of blood sampling in both native plastic tubes and containing ethylenediaminetetraacetic acid (EDTA). The tubes were centrifuged at 3000 g, and plasma was stored at –70°C until assayed.

DHEA (ng/mL) was measured by ELISA-Assay (IBL International GmbH, Hamburg, Germany). with an assay range between 0–30 ng/mL.For better comparison and calculation, DHEA values were converted to nmol/L.

DHEAS (micromol/L) was measured by ElectroChemiLuminescence (ECL) cobas 6000 analyzer (Roche Diagnostics, Basel, Switzerland) with an assay range between 0.003–27.0 micromol/L.

1 microgram ACTH-stimulation tests were performed using 0.25 mg tetracosactidum (0.250 mg/mL synthetic ACTH1–24, Synacthen©, Novartis Pharma, Switzerland) divided into 0.001 mg tetracosactidum doses by the pharmacy of the University Hospital Basel (Basel, Switzerland), as described [3], [44]. In all subjects, blood samples were taken at 0 min. for the basal measurement of cortisol and again at 30 min. for the measurement of serum cortisol concentration after intravenous (iv) administration of 1 microgram ACTH.

Cortisol was measured with a competitive chemiluminescence-immunoassay (IMMULITE 2000; Siemens Medical Solution Diagnostics, Los Angeles, USA), which has a sensitivity of 0.02 microgram/mL with an intra-assay coefficient of variation of 6.8% to 9.0%.

### Statistical Analysis

Discrete variables are expressed as percentage and continuous variables as medians and interquartile ranges (IQR) or as mean with standard deviation (SD) based on the distribution of the data. Two group comparisons of not normally distributed data were made using the Mann-Whitney U test. Spearman’s Rank Correlation was used for calculating correlations. For multi-group comparisons Kruskal-Wallis was used as a global test.

As a parameter of adrenal dissociation between cortisol and DHEA(S) synthesis, the ratios cortisol/DHEA and cortisol/DHEAS were calculated. To test whether the values of DHEA, DHEAS, cortisol, stimulated cortisol 30 minutes after ACTH, and delta cortisol are associated with poor functional outcome or survival, we calculated a logistic regression analysis adjusted by age, gender and comorbidities using the Charlson Index [45]. Time to last follow-up, death or re-event was analyzed in Kaplan-Meier curves. The three groups “good outcome” corresponding to an mRS of 0–2, “poor outcome but survival” corresponding to an mRS of 3–5 and “non-survival”, corresponding to an mRS of 6, were also compared.

Due to the known influence of age and gender on DHEAS and DHEA levels [46]–[48], we assessed the effects of age and gender. Age classes were defined by decades <60 y, 60–69 y, 70–79 y, and >80 y.

All testing was 2-tailed and the significance level was defined as p<0.05 or, if comparing 3 groups, p<0.016. For all calculations, STATA 9.2 (Stata Corp, College Station,TX, USA) and GraphPad Prism 5.0 (La Jolla, Ca, USA) were used.

## Results

### Baseline Characteristics

Of the original 362 ischemic stroke patients, 231 had both an available serum sample and a low-dose ACTH at day 1 after admission and were thus eligible for this analysis. Baseline characteristics of the analyzed and the original sample were similar (data not shown). The median NIHSS on admission was 5 (IQR 2–10). 37% (86 patients) had an unfavorable functional outcome (i.e. mRS >2) after 1 year, and all-cause-mortality after 1 year was 17.0% (38 patients). Baseline characteristics are shown in Table 1.

**Table 1:** Baseline characteristics.

Age, yr (mean ± SD)	71±14
Male sex, no (%)	137 (59%)
**Clinical findings**	mean ± SD
body temperature, °C	37.0±0.7
Heart rate, beats/min	78±16.69
systolic blood pressure, mmHg	161±26.24
diastolic blood pressure, mmHg	91±16
**Medication upon admittance**	
Diuretics	80 (35%)
ACE-inhibitors	53 (23%)
Sartans	43 (19%)
Ca-antagonists	31 (13%)
Betablockers	58 (25%)
Statins	50 (22%)
Phenprocoumon	21 (19%)
Acetyl salicylate 100–300 mg daily	86 (37%)
Benzodiazepines	27 (11.7%)
Glucocorticoids	9 (3.9%)
**Clinical Stroke Syndrome**	
TACS	22 (10%)
PACS	106 (46%)
LACS	48 (21%)
POCS	55 (24%)
**Scores**	median (IQR)
NIHSS on admission (pts)	5 (2–10)
Charlson Comorbidity Index (pts)	1 (0–2)
**Laboratory findings**	median (IQR)
Cortisol basal (nmol/L)	475 (337–611)
Cortisol 30 min. (nmol/L)	742 (619.5–859.5)
Δ Cortisol (nmol/L)	257 (156.5–376)
DHEA (nmol/L)	15.5 (9.4–25.0)
DHEAS (µmol/L)	2.4 (1.3–3.7)
**Outcome**	
death after 1 year, no. (%)	38 (16.45)
Lost to follow-up after 1 year, no. (%)	7 (3)

n = 231.

9 patients (3.9%) were on an oral glucocorticoid treatment on admission; their levels of DHEA, basal and stimulated cortisol were lower (p = 0.0027, 0.0267, 0.0423) as compared to patients without glucocorticoid treatment, whereas the levels of DHEAS (p = 0.0548), delta cortisol (p = 0.6) and the ratios cortisol/DHEA (p = 0.7) and cortisol/DHEAS (p = 0.9) were not different in patients with glucocorticoid treatment compared to patients without glucocorticoid treatment. 27 patients (11.7%) were on benzodiazepine medication; their levels of stimulated and delta cortisol were higher (p = 0.0053 and 0.0052), whereas DHEA (p = 0.8), DHEAS (p = 0.4), basal cortisol (p = 0.4) and the ratios cortisol/DHEA (p = 0.7) and cortisol/DHEAS (p = 0.1) did not differ between patients with and without benzodiazepine medication. 4 patients (1.7%) were on antiepileptic treatment, 4 (1.7%) had tricyclic antidepressants, 2 (0.9%) had opiate analgetics, whereas dopamine antagonists and haloperidol were taken by only one patient (0.4%).

Of the 131 patients with incomplete data that were not included in this analysis, 13 were lost to follow-up, 54% had an mRS >2 and thus an unfavourable outcome after 1 year, and mortality after 1 year in these patients was 22%. Lost to follow-up of the patients qualifying for this analysis was 3% or 7 patients.

Median baseline DHEA, DHEAS, basal cortisol, stimulated cortisol after 30 minutes and delta cortisol were 15.5 nmol/L (IQR 9.4–25.0), 2.4 umol/L (IQR 1.3–3.7), 475 nmol/L (IQR 337–611), 742 nmol/L (619.5–859.5) nmol/L, and 257 nmol/L (IQR 156.5–376), respectively.

### Gender and Age Differences

Overall gender differences were found in DHEA (p = 0.0047), which was higher in women, and DHEAS (p = 0.0002), which was higher in men. Neither basal (p = 0.4801), stimulated (p = 0.0572), delta cortisol (p = 0.5220) nor cortisol/DHEA (p = 0.8138) showed significant gender differences.

A decrease of DHEAS across age classes was found both in men (p<0.0001) and women (p = 0.0015), which lead to an increase of the cortisol/DHEAS - ratio across age classes (women: p = 0.0036, men: p<0.0001).

### Correlation between Stroke Severity and Adrenal Function

Basal and stimulated cortisol correlated with severity of stroke as assessed by NIHSS (correlation coefficient 0.36 (p<0.0001) and 0.32 (p<0.0001). The ratios cortisol/DHEA and cortisol/DHEAS showed a weaker correlation (correlation coefficient 0.16, p = 0.02 and 0.15, p = 0.02). There was no significant correlation between NIHSS and delta cortisol, as well as DHEA and DHEAS.

### Adrenal Function and Functional Outcome

Before adjusting for covariates, DHEAS, and to a lesser extent DHEA, were associated with functional outcome (p = 0.0212 and p = 0.0744). Basal and stimulated cortisol, but not delta cortisol, were also associated with functional outcome (p = 0.0013, 0.0389, and 0.4996, respectively). The ratios cortisol/DHEA and cortisol/DHEAS were also associated with functional outcome (p = 0.0047 and 0.0002).

After adjusting for age, gender and the Charlson Index, results remained largely unchanged except the ratios cortisol/DHEA and cortisol/DHEAS.

DHEAS, but not DHEA, was predictive for poor functional outcome (adjusted OR 1.21 (95% CI 1.01–1.49, and 1.01 (0.99–1.04), respectively). Basal cortisol remained predictive for functional outcome (adjusted OR 1.20 (95% CI 1.04–1.38), whereas stimulated cortisol and delta cortisol did not have any predictive value (adjusted OR 1.11 (95% CI 0.97–1.27 and 0.86 (0.71–1.05), respectively). The ratios cortisol/DHEA and cortisol/DHEAS were not predictive for functional outcome (adjusted OR for both 1.0 (95% CI 0.99–1.0).

For detailed values, see Table 2.

**Table 2:** Prognostic value of DHEA, DHEAS, basal cortisol, stimulated, delta cortisol, and the ratios cortisol/DHEA and cortisol/DHEAS for functional outcome after 1 year.

Functional outcome	mRS 2 or less,median (IQR)	mRS>2,median (IQR)	Adjusted* OR, (95% CI)
**DHEA(nmol/L)**	16.9(10.7–26.2)	14.0(8.4–23.2)	1.01(0.99–1.04)
**DHEAS(micromol/L)**	2.7(1.2–4.0)	2.0(1.3–2.8)	**1.23(1.01–1.49)**
**Cortisol(nmol/L)**	452.5(323–569)	511.5(372.3–680)	**1.20(1.04–1.38)**
**Stimulated Cortisol(nmol/L)**	731(623–837)	774(641–911)	1.11(0.97–1.27)
**Delta cortisol(nmol/L)**	267.5(169.8–384)	247.5(141–374)	0.86(0.71–1.05)
**Cortisol/DHEA (nmol/nmol)**	24 (16–35)	31 (19–60)	1.0 (0.99–1.00)
**Cortisol/DHEAS (nmol/micromol)**	171 (113–296)	248 (169–433)	1.0 (0.99–1.00)

* adjusted for gender, age and charlson index.

### Adrenal Function and Mortality

Before adjusting for covariates, DHEAS, but not DHEA, was associated with all-cause-mortality after 1 year (p = 0.0709 and 0.0477, respectively). Basal and stimulated cortisol, but not delta cortisol, were also associated with mortality (p<0.0001, p = 0.0013, and p = 0.3232, respectively). The ratios cortisol/DHEA and cortisol/DHEAS were associated with mortality as well (p = 0.0001 and 0.0003).

After adjusting for age, gender and the Charlson Index, neither DHEA nor DHEAS remained predictive for mortality (adjusted OR 0.99 and 1.10 (95% CI 0.96–1.03 and 0.82–1.44). Basal and stimulated cortisol, but not delta cortisol, were predictive for mortality (adjusted OR 1.41 (95% CI 1.20–1.71), 1.35 (95% CI 1.15–1.60), and 0.92 (95% CI 0.72–1.17), respectively.) The ratios cortisol/DHEA and cortisol/DHEAS were not predictive for mortality (adjusted OR for both 1.0 (95% CI 0.99–1.0 and 1.0–1.01, respectively). For detailed results, see Table 3.

**Table 3:** Prognostic value of DHEA, DHEAS, basal cortisol, stimulated cortisol, delta cortisol, and the ratios cortisol/DHEA and cortisol/DHEAS for mortality after 1 year.

Mortality	survivors, median (IQR)	Nonsurvivors, median (IQR)	Adjusted* OR, (95% CI)
**DHEA (nmol/L)**	15.9 (9.9–25.5)	12.1 (8.2–22.8)	0.99 (0.96–1.03)
**DHEAS (micromol/L)**	2.5 (1.3–3.9)	1.9 (1.2–2.8)	1.10 (0.82–1.44)
**Cortisol (nmol/L)**	461 (325–585)	606 (471–800)	1.41 (1.20–1.71)
**Stimulated Cortisol (nmol/L)**	728 (618–841)	836 (691–1078)	1.35 (1.15–1.60)
**Delta cortisol (nmol/L)**	261 (164–380)	241 (139–369)	0.92 (0.72–1.17)
**Cortisol/DHEA (nmol/nmol)**	16 (16–37)	190 (117–304)	1.0 (1.0–1.01)
**Cortisol/DHEAS (nmol/micromol)**	46 (27–78)	389 (233–582)	1.0 (0.99–1.00)

* adjusted for gender, age and charlson index.

**Figure 1:**
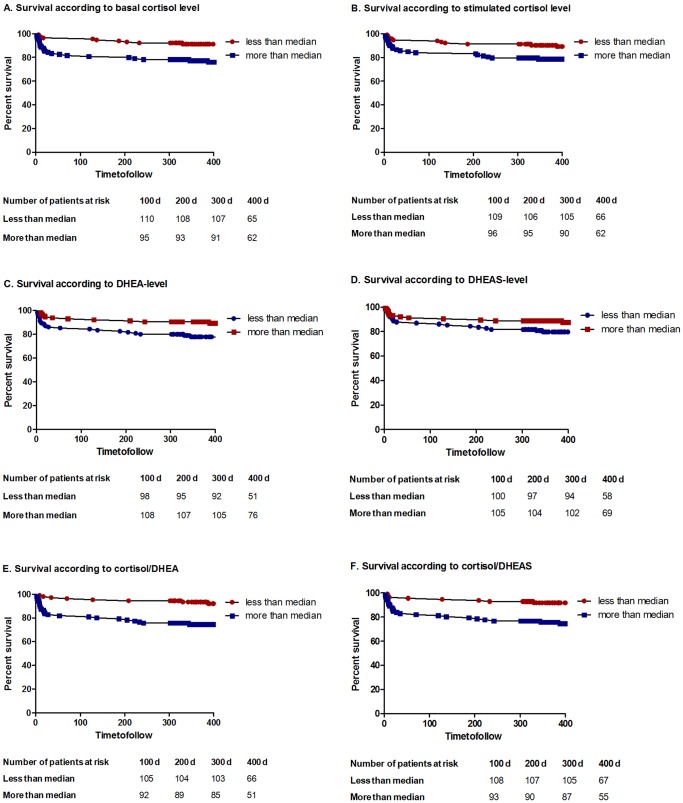
Kaplan Meier Survival Curves. A. Survival in relation to median cortisol level of 475 nmol/L. p = 0.0014, Hazard Ratio 0.35, 95% CI 0.19–0.67. B. Survival in relation to median stimulated cortisol level of 742 nmol/L. p = 0.015, Hazard Ratio 0.4496, 95% CI 0.24–0.86. C. Survival in relation to median dehydroepiandrosterone level of 15.5 nmol/L. p = 0.0236, Hazard Ratio 2.09, 95% CI 1.104 to 3.955. D. Survival in relation to median dehydroepiandrosterone-sulfate level of 2.4 umol/L. p = 0.1490, Hazard Ratio 1.60, 95% CI 0.85–3.80. E. Survival in relation to median cortisol/DHEA level of 27 nmol/nmol. p = 0.0002, Hazard Ratio 0.32, 95% CI 0.17–0.61. F: Survival in relation to median cortisol/DHEAS level of 203 nmol/micromol. p = 0.0004, Hazard Ratio 0.31, 95% CI 0.17–0.59.

Overall survival was better in patients with a basal cortisol level below the median of 475 nmol/L (p = 0.001, Hazard Ratio (HR) 0.35, 95% CI 0.19–0.67; Figure 1A) and with a stimulated cortisol level below the median of 742 nmol/L (p = 0.02, HR 0.45, 95% CI 0.24–0.86, Figure 1B). Overall survival was better in patients with a DHEA level above the median level of 15.5 nmol/L (p = 0.0236, Hazard Ratio 2.09, 95% CI 1.104 to 3.955; Figure 1C). DHEAS survival was not significant (p = 0.1490, Hazard Ratio 1.60, 95% CI 0.85–3.80; Figure 1D). Overall survival was better in patients with a cortisol/DHEA ratio below the median of 27 nmol/nmol (p = 0.0002, Hazard Ratio 0.32, 95% CI 0.17–0.61; Figure 1E) and with a cortisol/DHEAS ratio below the median of 203 nmol/micromol (p = 0.0004, Hazard Ratio 0.31, 95% CI 0.17–0.59; Figure 1F).

**Figure 2:**
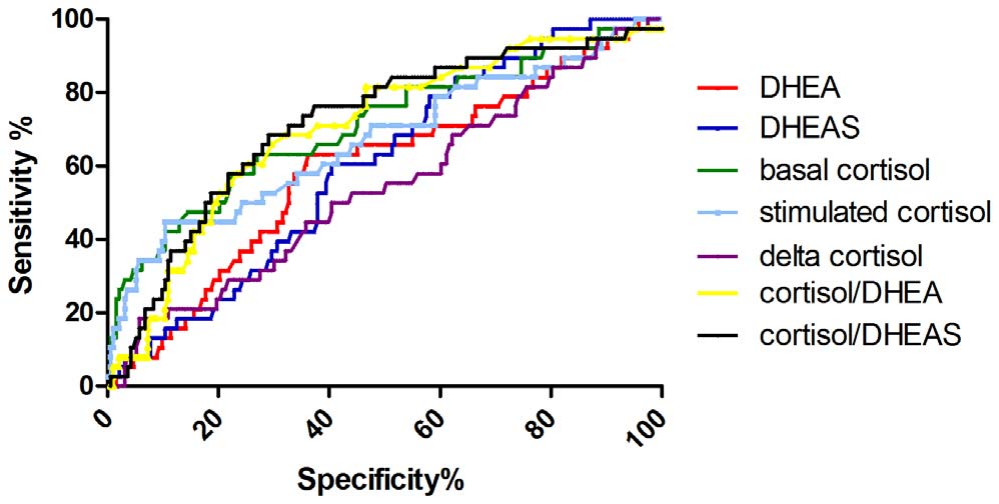
ROC of adrenal values. Dehydroepiandrosterone (DHEA): Area 0.59, Std. Error 0.05, 95% CI 0.49–0.69, p = 0.07 Dehydroepiandrosterone-sulfate (DHEAS): Area 0.60, Std. Error 0.04, 95% CI 0.51–0.69, p = 0.048. Basal cortisol: Area 0.71, Std. Error 0.05, 95% CI 0.61–0.80, p<0.0001. Stimulated cortisol: Area 0.67, Std. Error 0.05, 95% CI 0.57–0.77, p = 0.001. Cortisol/DHEA: Area 0.70, Std. Error 0.05, 95% CI 0.61–0.79, p<0.0001. Cortisol/DHEAS: Area 0.71, Std. Error 0.05, 95% CI 0.63–0.81, p<0.0001.

The area under the curve (AUC) was best for basal cortisol and cortisol/DHEAS with 0.71 (95% CI 0.61–0.80 for basal cortisol and 0.63–0.81 for cortisol/DHEAS), followed by cortisol/DHEA (AUC 0.70, 95% CI 0.61–0.79), stimulated cortisol (AUC 0.67, 95% CI 0.57–0.77), DHEAS (AUC 0.60, 95% CI 0.51–0.69), and DHEA (AUC 0.59, 95% CI 0.49–0.69) (see figure 2).

### “Poor Outcome but Survival” Compared to Non-survival

We found an overall difference between the groups mRS 0–2 (“good outcome”), mRS 3–5 (“poor outcome but survival”) and mRS 6 (“non-survival”) for basal cortisol (p = 0.0002), stimulated cortisol (p = 0.0041) and the ratios cortisol/DHEA (p = 0.0003) and cortisol/DHEAS (p<0.0001).

For basal cortisol, there was only a difference between mRS 0–2 and mRS 6 (p = 0.0010) but not between mRS 3–5 and mRS 6.

For stimulated cortisol, a difference was found between mRS 0–2 and mRS 6 (p = 0.0010) and between mRS 3–5 and mRS 6 (p = 0.0112).

For the ratio cortisol/DHEA and cortisol/DHEAS, we found differences between mRS 0–2 and mRS 6 (both p<0.0001) and between mRS 3–5 and mRS 6 (p = 0.0071 and p = 0.0029).

### Clinical Stroke Syndrom, Lesion Size and Andrenal Parameters

Values of basal cortisol showed significant differences with highest values in TACS and lowest in PACS (p = 0.002); stimulated cortisol was highest in PACS, lower in LACS (p = 0.0002) and lowest in POCS (p = 0.0002). DHEA, DHEAS and their ratios with cortisol did not depend on the subtype of stroke according to the Oxfordshire Community Stroke Project criteria (all p>0.1).

Lesion size correlated with basal (correlation coefficient 0.36, p = 0.0361) and stimulated cortisol (correlation coefficient 0.32, p = 0.0027) and cortisol/DHEA (correlation coefficient 0.15, p = 0.0289) as well, but not with delta cortisol (p = 0.28), DHEA (p = 0.27), DHEAS (p = 0.26), or cortisol/DHEAS (p = 0.74).

## Discussion

Acute ischemic stroke activates the HPA-axis [6], [49], and the role of adrenal function as outcome predictor is debated [5], [6], [36], [38], [39].

In our study, DHEAS but not its precursor DHEA was associated with functional outcome, but not with survival. The different result for DHEA and DHEAS is most likely due to the short serum half-life, and the pronounced intraindividual and interindividual variability of DHEA.

Previous observations in patients with community-acquired pneumonia, septic shock, critical illness and trauma found a predictive value of high DHEAS levels for both favorable [14]–[16], [50] and unfavorable outcome [15], [16], [51]. So far, only two studies investigated DHEAS levels in stroke, again with controversial findings. One study did not find any correlation between DHEAS levels and functional outcome [5], whereas the other found a significant association with functional outcome but not with mortality in a population of postmenopausal women [36]. In some, but not all [52] epidemiologic studies, low DHEAS-levels were predictive for intima-media-thickness [53], [54], cardiovascular disease [28], [55] and all-cause mortality [29], [56], [57]. In rats, DHEA has been shown to have a direct local neuroprotective effect in ischemia if administered in the right time window [58], indicating a local neuroprotective effect of systemic DHEA/−S. Low DHEA/−S levels may therefore represent a lack of neuroprotection and/or vascular protection.

Although acute ischemic stroke is considered an acute critical illness, findings from other critical illnesses concerning DHEA and DHEAS may not translate into the field of ischemic stroke. DHEA and DHEAS are also synthesized locally in the brain [23] and peripheral DHEA- and DHEAS-levels may be influenced by brain DHEA/−S synthesis [59], [60].

In our patients, we found that low DHEAS levels predicted adverse functional outcome. This finding is in agreement with the assumed neuroprotective role of DHEAS in ischemic stroke[20]–[22]. It may be that lower DHEAS synthesis leads to a higher ischemic neuronal loss after stroke and thus unfavorable outcome. Our data showing a significant association of DHEAS and outcome can, however, not answer the question whether lower DHEAS is a cause or a consequence of ischemic stroke, or a combination of both. An increased cortisol/DHEAS ratio has been identified as a risk factor for atherosclerosis [26], [27]. Therefore, DHEAS and cortisol/DHEAS ratio upon admittance may represent the values to which the patient was exposed before stroke pointing to a potential causal factor of stroke. On the other hand, DHEAS and cortisol/DHEAS ratio may represent the acute activation of the HPA-axis during or after stroke and could therefore be the consequence of the stroke.

In accordance with previous literature, basal cortisol is a predictor for mortality and functional outcome in patients with ischemic stroke in our cohort, with higher levels in patients with an unfavorable outcome [5]–[9], [38]. High basal cortisol reflects an activated HPA-axis and a higher stress level. Stimulated cortisol is a measure of adrenal reserve. In our study, basal cortisol was a stronger predictor for outcome and mortality than stimulated cortisol levels.

As basal and stimulated cortisol levels are correlated with the NIHSS, it is suggestive that the amount of activation of the HPA-axis is at least partially a result of the acute ischemic event. This is also supported by the correlation of lesion size with cortisol values. Furthermore, delta cortisol was not associated with functional outcome or mortality. Most probably, this is due to the high variability of the cortisol surge and due to the fact that some of the patients are already maximally stimulated by physical stress [61]. A critical illness-related corticosteroid insufficiency (CIRCI), as described and controversially debated in intensive care patients [62], is defined by a relatively low basal cortisol or an insufficient increase after ACTH stimulation and associated with a higher mortality in septic shock [63], [64]. According to our data, the amount of cortisol increase upon ACTH stimulation seems not to be relevant and predictive in stroke. This is in accordance with data of ACTH stimulation in severe pneumonia [65]. Whereas early activation of the HPA-axis at a hypothalamic level has been shown to be predictive for functional outcome and survival in acute ischemic stroke and is easily measurable [8], [66], the adrenal reaction is more subtle. The prognostic value of DHEAS and cortisol for functional outcome shows that acute ischemic stroke with unfavorable outcome is a state in which the HPA-axis and the adrenals as its end-organs react with a relatively higher synthesis of cortisol in comparison to DHEAS, leading to a higher cortisol/DHEAS ratio than in patients with favourable outcome. At the level of the adrenals, this means a shift from synthesis of androgen precursor hormones, which the body apparently does not need for imminent survival, to synthesis of a stress hormone thought to be essential for survival in acute illness. Accordingly, there was a gradual increase of the ratios cortisol/DHEA and cortisol/DHEAS from good outcome to poor functional outcome to non-survival. The ratio cortisol/DHEAS had the same AUC like basal cortisol (see figure 2) and a lower hazard ratio (see figure 1), being as sensitive for mortality in acute ischemic stroke as basal cortisol in univariate but not multivariate analysis.

An increased cortisol/DHEAS ratio has been found to be a surrogate for neurodegenerative diseases[31]–[33]. Therefore, an alternative hypothesis is that the cortisol/DHEAS ratio may represent a slightly lower cerebral DHEAS production in relation to cortisol already before the stroke in predisposing patients. Although we found a predictive value for outcome and mortality for the cortisol/DHEAS-ratio in univariate analysis, it did not remain after adjustment, moreover DHEAS itself was only predictive for outcome and not for survival.

There are several limitations to this study.

First, we performed single measurements of adrenal hormones upon admission to the hospital. This bears a potential for bias, as cortisol, DHEA, DHEAS and the adrenal reactivity to the ACTH test has a certain diurnal and individual variability and may change fast in relation to external stimuli. Repeated measurements and urinary collection may further improve the prognostic ability of hormones for outcome prediction. Yet, previous studies have shown that initial hormone measurements predict outcome in acute illness and are easier to collect on an everyday basis [67], [68], whereas in chronic illnesses and neurodegenerative processes [69]–[72], measurement of urinary excretion of steroid metabolites has proven to be more feasible and useful.

Second, we did not collect specimen in patients before stroke onset. Therefore, we cannot differentiate between baseline abnormalities and acute changes due to the event itself.

Third, a second measurement of adrenal hormones at the time of follow-up would have permitted some insights in the long term dynamics of these markers. However, this has not been performed due to limited resources.

Fourth, with our data, it is not possible to discriminate between adrenal and cerebral synthesis of DHEA. Since in stroke patients we do not routinely perform spinal taps we have no means of easily assessing potential possible intra-cerebral production of DHEAS.

Finally, whereas we collected detailed data on drugs which are known to be associated with stroke prognosis like antihypertensive drugs and statins, we have only limited knowledge on medication with potential influence on adrenal function. However, by adjusting to the Charlson Index, the data is adjusted to comorbidities like dementia, diabetes, hemato-oncologic diseases and connective tissue disease that have the potential to influence adrenal function directly or via concomitant medication.

## Conclusions

This study found that surrogates of adrenal androgen and glucocorticoid production are predictive for functional outcome and mortality in acute ischemic stroke. DHEAS is predictive for functional outcome only, whereas cortisol levels are predictive of both, functional outcome and mortality. An increased cortisol/DHEAS ratio is associated with an unfavourable outcome, in uni- but not in multivariate analysis. The low-dose-ACTH-test does not add any additional prognostic information. CIRCI, a term controversially discussed in critical illness, is apparently not of clinical relevance in acute stroke.
